# Prevalence of Urinary Tract Infections and Antibiogram of Bacteria Isolated From Children With Sickle Cell Disease in Tanzania

**DOI:** 10.7759/cureus.58786

**Published:** 2024-04-22

**Authors:** Raphael Z Sangeda, Joseph Yohana, Agnes Jonathan, Vicky P Manyanga, Deogratius Soka, Julie Makani

**Affiliations:** 1 Department of Pharmaceutical Microbiology, Muhimbili University of Health and Allied Sciences, Dar es Salaam, TZA; 2 Sickle Cell Program, Muhimbili University of Health and Allied Sciences, Dar es Salaam, TZA; 3 Department of Medicinal Chemistry, Muhimbili University of Health and Allied Sciences, Dar es Salaam, TZA; 4 Haematology, Tanzania Sickle Cell Disease Alliance, Dar es Salaam, TZA; 5 Department of Haematology and Blood Transfusion/Sickle Cell Program, Muhimbili University of Health and Allied Sciences, Dar es Salaam, TZA

**Keywords:** children, amr, uti, scd, sickle cell disease

## Abstract

Introduction

Individuals with sickle cell disease (SCD) are particularly vulnerable to urinary tract infections (UTIs) due to immunological deficits and renal abnormalities associated with the disorder. These infections can exacerbate underlying health issues and lead to severe complications if not managed promptly and effectively. Due to the heightened risk and potential consequences of UTIs in this population, this study aimed to determine their prevalence and explore the resistance patterns of causative pathogens among children attending the SCD Clinic at Muhimbili National Hospital (MNH), Dar es Salaam, Tanzania. Focusing on this demographic group, we sought to provide targeted insights to inform better clinical protocols and intervention strategies in regions heavily affected by SCD.

Materials and methods

This prospective cross-sectional study was conducted at the MNH, Dar es Salaam, Tanzania, with an enrollment over two months from 19^th^ March to 21^st^ May 2015. We diagnosed UTIs in children with SCD using dipstick and culture methods. Antibiotic susceptibility was assessed using the Kirby-Bauer disc diffusion method, evaluating resistance patterns to antibiotics such as ampicillin, cloxacillin, erythromycin, chloramphenicol, ceftriaxone, and trimethoprim-sulfamethoxazole. The diagnostic accuracy of the dipstick and culture methods was validated to ensure reliability in detecting UTIs. Statistical analysis was conducted using Statistical Product and Service Solutions (SPSS) software (Released 2019; IBM Corp., Armonk, New York, United States).

Results

Among the 250 children, 56 (22.4%) were UTI-positive according to the culture method and 62 (24.8%) were UTI-positive according to the dipstick test. Girls were more likely to be UTI-positive than boys (29.1% and 13.6%, respectively; p-value = 0.011). *Escherichia coli *was the most common uropathogen, followed by *Klebsiella, Staphylococcus, Proteus, *and *Pseudomonas *(44.2%, 26.9%, 21.2%, 3.8%, and 1.9%, respectively). All isolates were resistant to ampiclox. Resistance rates to ampicillin, erythromycin, cotrimoxazole, chloramphenicol, and ceftriaxone were 94.2%, 76.9%, 59.6%, 46.2%, and 21.2%, respectively.

Conclusion

This study indicated that dipsticks diagnosed more UTIs. The prevalence was higher in girls than in boys. *Escherichia coli* was the most commonly isolated antibiotic-resistant organism. High resistance levels were observed against the combination of ampicillin and cloxacillin. However,* *the isolates were less resistant to ceftriaxone. These results call for increased surveillance of resistant uropathogens in the pediatric population with SCD.

## Introduction

Sickle cell disease (SCD) is a hereditary disorder that results in abnormalities in oxygen-carrying hemoglobin molecules in the red blood cells. This causes the cells to assume an abnormal, rigid, and sickle-like shape. Consequently, individuals with SCD experience several acute and chronic health complications such as severe infections, frequent severe pain, stroke, and an increased risk of death [1]. The American physician James B. Herrick first described this condition in the medical literature in 1910 [2].

Tanzania has one of the highest annual births of SCD-affected individuals globally, estimated to be between 8,000 and 11,000 births per year [3]. Moreover, more than 10,000 children under five years of age are estimated to die annually, contributing to an estimated 7% of the overall deaths of children in this population [4].

Interventions, such as newborn screening and enrollment in comprehensive care programs involving prompt diagnosis and treatment of complications and prevention of infections by vaccination and oral penicillin, have markedly improved the survival of patients with SCD in Europe and America. Unfortunately, the introduction of interventions in Africa has been hampered by a lack of locally obtained and relevant evidence [5].

Urinary tract infections (UTIs) are more common in African children of the same age with SCD than in children without SCD [6-9]. Its incidence has also been reported in children living in the USA, with a diagnosis rate of 4% [9]. UTI affects the quality of life of SCD patients. UTI results from splenic impairment in this group of patients and is a significant cause of childhood morbidity [6]. It is a simple form of cystitis that involves the lower urinary tract (bladder infection). It is also known as pyelonephritis (kidney infection) and affects the upper urinary tract. Lower urinary tract symptoms included painful urination, frequent urination, and urination (or both). In contrast, patients with pyelonephritis have fever and flank pain, in addition to the symptoms of a lower UTI [10]. Individuals with SCD are at an increased risk [11] because delays in inappropriate treatment of UTI can result in scar formation and subsequent renal impairment [12,13].

Knowledge of the sensitivity patterns of common bacteria that cause UTIs is essential for selecting appropriate medications to help prevent fatal complications, such as renal impairment. The following were observed in a study conducted at Muhimbili National Hospital on febrile children. Among the 382 children, sixty-four were confirmed to have UTIs by urine culture, with an overall prevalence of 16.8%. Among the isolated uropathogens, 79.7%, 89.1%, and 70.3% were resistant to ampicillin, cotrimoxazole, and clavulanate-potentiated amoxicillin, respectively, and 54.7% and 50% were resistant to gentamycin and ceftriaxone, respectively. Amikacin exhibited the lowest resistance rate (12.5%) [5].

Tanzania is among the most malaria-endemic areas, similar to other African countries. In malaria-endemic regions, fever is the main diagnostic predictor of malaria, leading to misdiagnosis of various conditions caused by fever, particularly in children [14]. For instance, in a study of non-malarial causes of fever, the most frequent cause was viral infection (52.6%), followed by UTI (21.6%). Other types of fever included skin infections (11.6%), bacteremia (2.7%), otitis media (2.6%), and measles (2.1%) [14].

According to the Tanzania standard treatment guidelines at the time of this study, the antibiotics recommended to treat UTIs in complicated cases were ciprofloxacin, amoxicillin/clavulanic acid, nitrofurantoin, benzathine, penicillin, erythromycin, azithromycin [15], and cephalosporins such as ceftriaxone [15]. However, there is a lack of studies reporting the prevalence of uropathogens among children with SCD, resistance profiles, and the diagnostic accuracy of UTI tests.

Given the high number of individuals born with SCD in Tanzania, there is a need to monitor and control patients at high risk of complications associated with UTI, such as hypertension and other kidney-related diseases, including renal failure, by providing them with an understanding of the epidemiology of UTI in SCD, thus providing appropriate care and management. Therefore, this study aimed to delineate the bacterial sensitivity landscape of uropathogens implicated in UTIs among Tanzanian children with SCD, identify prevalent microorganisms, assess their antibiotic susceptibility, and compare the prevalence and diagnostic efficacy of dipstick and culture methods.

## Materials and methods

Study design and setting

This prospective cross-sectional study was conducted as part of the Muhimbili Sickle Cell Cohort (MSC) initiative at the Sickle Cell Clinic of Muhimbili National Hospital (MNH) in Dar es Salaam, Tanzania, aimed at addressing SCD in the pediatric population aged 0-15 years. Dar es Salaam is a bustling city in Tanzania, home to approximately 5.38 million people in a country with a population exceeding 61 million [16]. MSC, initiated in 2004, represents a collaborative effort between the MNH and Muhimbili University of Health and Allied Sciences (MUHAS) to methodically explore the clinical spectrum of SCD, identify the causes of morbidity and mortality, and craft specific interventions for diagnosed individuals. Of the 8,484 individuals screened in the MSC program up to 2016, 5,476 were diagnosed with SCD. The clinic, catering to 300 to 350 patients monthly, collects comprehensive demographic data, revealing a participant gender distribution of 48.9% female and 51.1% male. Age-wise, 41.9% of participants were between 0 and 4 years old and 45.4% were between 5 and 17 years old, underscoring the study's focus on young individuals affected by SCD [17].

Study participants

Children with sickle cell disease were admitted to the MNH in Dar es Salaam, Tanzania, from 19th March to May 21st, 2015.

Sample size

Sample size calculation was done per the method used for medical studies [18]. The sample size was calculated using the formula:

$N = \frac{Z^2 \times (1 - P)}{E^2}$

where n is the sample size, z = Z score of 95%, a 1.96 confidence interval, P is the proportion from the previous study, and E= margin of permissible error, which is 5%.

A prevalence of 21.6% was observed in a similar study conducted in Nigeria in 2003 among febrile children with SCD admitted to the Ibadan Hospital [8]. Therefore, this study evaluated 260 children with SCD using this calculation.

$N = \frac{(1.96)^2 \times (100 - 21.6)}{5^2} = 260$

Based on the formula and parameters outlined above, our initial calculation targeted a sample size of 260 children with SCD for evaluation. However, due to practical constraints encountered during the recruitment phase, we successfully recruited only 250 participants. It is important to note that this recruited sample size still surpasses the participant count of 171 children in the referenced study [8], providing a substantial basis for comparison and analysis.

Data collection

Sample Collection and Preparation

Mid-stream urine, the widely recommended urine sampling method in children under five, where guardians were instructed to collect a urine sample in sterile conditions and containers, was used to collect urine samples from the children attending the SCD clinic at MNH. When the container was one-third complete, the lid was closed.

Samples were immediately tested using the dipstick method, and the results were recorded. After the dipstick analysis, the Clean, catch mid-stream urine samples were stored in a cool box and transported to the Pharmaceutical Microbiology laboratory for analysis.

Study Variables

A unique sample number was assigned to each test sample for identification. Demographic information, including Age and Sex, was collected to contextualize patient data. Fever presence, symptom duration before testing, and the use of antibacterial medication were documented. Urinalysis measures, such as leukocyte, nitrate, protein levels, urine pH, and specific gravity, were evaluated. The UTI Inference Dipstick test was conducted for initial screening and complemented by culture results to assess the bacteria. Isolation of any organism was noted, and the cause of the infection was identified. Furthermore, when an organism was identified, its sensitivity to various antibiotics was recorded to aid in selecting the most effective treatment.

Culture and Identification

The samples were subsequently cultured in cysteine lactose electrolyte-deficient (CLED) agar medium using a calibrated sterile wire loop. The remaining samples were stored at four centigrade. After overnight culture, the significant growth was further analyzed, and the bacteria were isolated and identified through colony morphology and biochemical tests. More than 1×10^5^/mil colony-forming units (CFU)/ml was required for growth to be statistically significant. The CFU were calculated by comparing the volume delivered by a wire loop of approximately 15 μL on the plate compared to the numbers in 1 mL tubes. Growth of ≥1 ×10^5^ CFU/ml indicated significant growth.

Antimicrobial Susceptibility Testing

The Kirby-Bauer disc diffusion method was used according to the Clinical and Laboratory Standards Institute (CLSI) guidelines (2015) for antimicrobial susceptibility testing (AST) [19]. Nutrient agar was used to culture bacterial colonies during analysis, and the samples were stored in a refrigerator. Finally, the AST of the strains of ampicillin, ampiclox (ampicillin and cloxacillin), erythromycin, chloramphenicol, ceftriaxone, and cotrimoxazole (trimethoprim and sulfamethoxazole) was determined using commercial discs. The AST results of the clinical isolates were validated using standardized control strains of* Escherichia coli *(ATCC 25922), *Staphylococcus aureus* (ATCC 25923), *Klebsiella pneumoniae* (ATCC 13883),* Proteus mirabilis *(ATCC 25933) and *Pseudomonas aeruginosa *(ATCC 27853). AST was determined after diluting the bacteria suspension in comparison to McFarland standard solution. Mueller-Hinton agar was used as the medium for AST. Inhibition zones were measured and compared with those of every antibiotic tested, and the results were recorded as sensitive, intermediate, or resistant. The antibiotics tested were ampicillin, erythromycin, ampiclox (ampicillin and cloxacillin), chloramphenicol, ceftriaxone, and cotrimoxazole (trimethoprim and sulfamethoxazole).

Data analysis

Statistical analysis was conducted using Statistical Product and Service Solutions (SPSS) software version 26 (IBM SPSS Statistics, IBM Corp., Armonk, NY, USA), where demographic and laboratory data were synthesized into charts and tables to represent variable proportions. The Chi-square test was used to determine the significance of the differences between gender, age and UTI status. The diagnostic accuracy of the dipstick test was evaluated by calculating the sensitivity and specificity with a 2 × 2 contingency table using culture results as the benchmark "gold standard." Sensitivity, the measure of the precision of the test in identifying actual UTI cases, was calculated using the formula

$\text{Sensitivity} = \frac{TP}{TP + FN}$

whereas specificity or the ability of the test to correctly rule out the absence of UTIs was calculated using the formula $\text{Specificity} = \frac{TN}{TN + FP}$

Where: TP = True Positives; number of patients with positive UTI by culture and tested positive on the dipstick; TN = True Negatives; number of patients with negative UTI by culture who also tested negative on the dipstick; FP = False Positives; number of patients with negative UTI by the culture method but tested positive on the dipstick; FN = False Negatives are patients with UTI positive results by culture but tested negative on the dipstick.

Ethical consideration

Ethical approval for this study was granted by the MUHAS Institutional Review Board, with approval number DA.25/111/01/March2015. Authorization to conduct the study was also obtained from the Muhimbili National Hospital. Participation was contingent upon informed consent from the parents or caregivers of children attending SCD clinics. Participants were required to provide midstream urine samples following detailed instructions to ensure sample integrity. The delay in publication until 2024 was primarily due to the unavailability of funds to support the publication process.

## Results

A total of 250 pediatric patients with SCD were enrolled in this study. The gender distribution was predominantly male, with 133 boys accounting for 53.2% of the participants. The age distribution showed that most children were between 11-15 years old, comprising 38% of the study population (Table 1). Fever, a common symptom of UTI, was reported in 16.4% (41 children), while the majority (83.6%) did not experience fever. Among those who reported fever, the most common duration was four days, observed in 31.7% of cases (Table 1). Regarding laboratory findings, bacterial growth was observed in 20.8% of SCD pediatric population samples, whereas 79.2% showed no organism growth. The colony-forming unit count was significant in 16.4% of the participants, aligning with the number of patients who reported fever (Table 1). Regarding treatment, 24% of the children were taking antibacterial medication. Penicillin V was the most frequently encountered antibiotic, accounting for 20.4% of participants (Table 1). Urinalysis revealed that 25.7% of the samples were positive for leukocytes, which is indicative of infection, whereas all samples tested negative for proteins. The urine pH varied, with the majority being 58.1%, with a pH of 6.5. The specific gravity of the urine was 1.030 in 68.7% of the samples.

**Table 1:** Social demographics characteristics, bacterial culture and urinalysis results for children with sickle cell disease (N=250)

Specific Variable (n = 250)	N (%)
Gender (n = 250)	
Boys	133 (53.2)
Girls	117 (46.8)
Age group	
0-5	66 (26.4)
6-10	89 (35.6)
11-15	95 (38.0)
Reported fever symptoms (n = 250)	
Yes	41 (16.4)
No	209 (83.6)
Duration of fever and days (n = 41)	
3	9 (22.0)
4	13 (31.7)
5	10 (24.4)
6	6 (14.6)
7	3 (7.3)
Organism was isolated(n = 250)	
Yes	52 (20.8)
No	198 (79.2)
Colony forming unit count (n = 250)	
Significant (>=1 x 10^5^)	41 (16.4)
Non-significant (<1 x 10^5^)	209 (83.6)
Antibacterial taken (n = 250)	
None	190 (76.0)
Amoxicillin	6 (2.4)
Amoxicillin + Penicillin V	1 (0.4)
Ampiclox	1 (0.4)
Azithromycin	1 (0.4)
Penicillin V	51 (20.4)
Leucocytes (n = 249)	
Positive	64 (25.7)
Negative	185 (74.3)
Proteins (n = 249)	
Negative	249 (100.0)
PH of urine (n = 248)	
6.0	79 (31.9)
6.5	144 (58.1)
7.0	24 (9.7)
8.0	1 (0.4)
Specific gravity of urine (n = 249)	
1.025	78 (31.3)
1.030	171 (68.7)

The prevalence of bacterial isolation was significantly higher in girls (29.1%) than in boys (15.8%) (p-value = 0.011) (Table 2). Looking at the age groups, the study showed a slightly non-significant increasing trend in bacterial isolation with age: 19.7% for ages 1-5, 22.5% for ages 6-10, and 23.2% for ages 11-15. years (p-value = 0.865), respectively (Table 2).

**Table 2:** Culture results and social demographics

Specific variable	Culture positive		Chi-square(p-value)
	Yes N (%)	No N (%)	
Age group			
1-5	13 (19.7)	53 (80.3)	0.290 (0.865)
6-10	20 (22.5)	69 (77.5)	
11-15	22 (23.2)	73 (76.8)	
Gender			
Boys	21 (15.8)	112 (84.2)	6.288 (0.011)
Girls	34 (29.1)	83 (70.9)	

**Figure 1:**
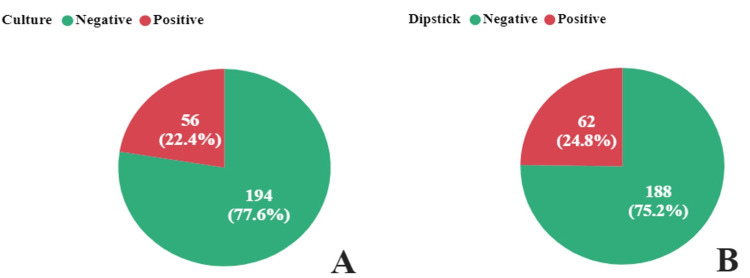
Patient diagnoses using culture and Dipstick methods The proportion of patients diagnosed with urinary tract infections among sickle cell children by culture methods (A) compared to the dipstick test (B).

According to the culture and dipstick tests, the prevalence of UTIs among children with SCD was 22.4% and 24.8%, respectively (Figure 1).

Specificity and sensitivity of dipstick

The sensitivity and specificity of the UTI inference dipstick test were 17.7% and 84.0%, respectively (Table 3).

**Table 3:** Specificity and sensitivity of dipstick versus culture diagnosis of urinary tract infection in children with sickle cell. *Key*: True Positives (TP) were patients correctly identified by both dipstick tests as having a UTI, which was confirmed by culture (n =11). True Negatives (TN) were correctly identified by the dipstick test as negative UTI, a finding that was also confirmed by culture (n =158). False Positives (FP) were incorrectly identified by the dipstick test as positive UTI when the culture was negative (n = 30). False Negatives (FN) were those with UTI, confirmed positive by culture but negative by dipstick (n = 51).

	Culture diagnosis		
Dipstick diagnosis	Negative	Positive	Dipstick total
Negative	158	30	188
Positive	51	11	62
Culture total	209	41	250

**Figure 2:**
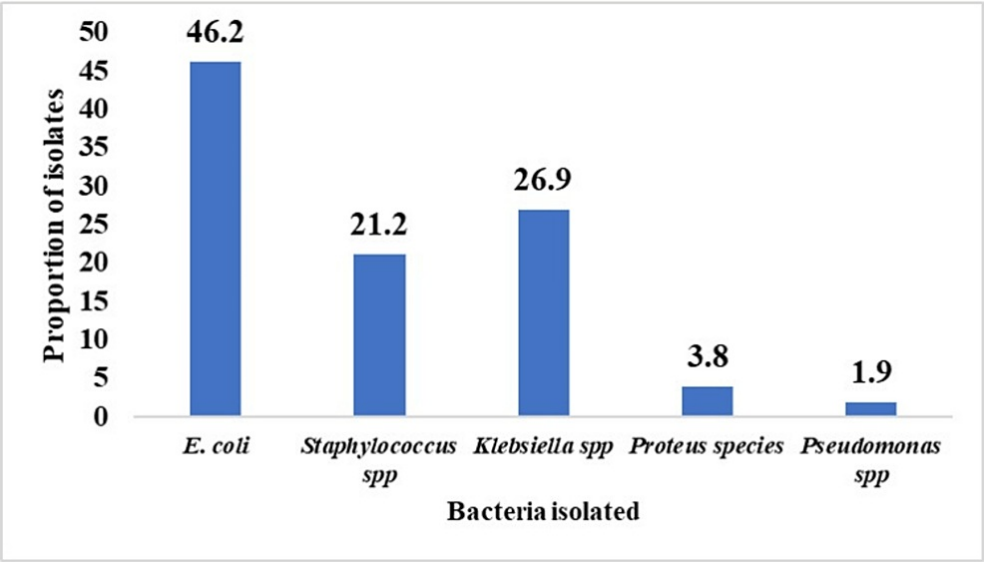
Proportion of isolated microorganisms associated with UTI (n =52). UTI, urinary tract infection

Most isolates from 52 children showed a predominance of *Escherichia coli*, with an isolation rate of 46.2% (Figure 2).

**Figure 3:**
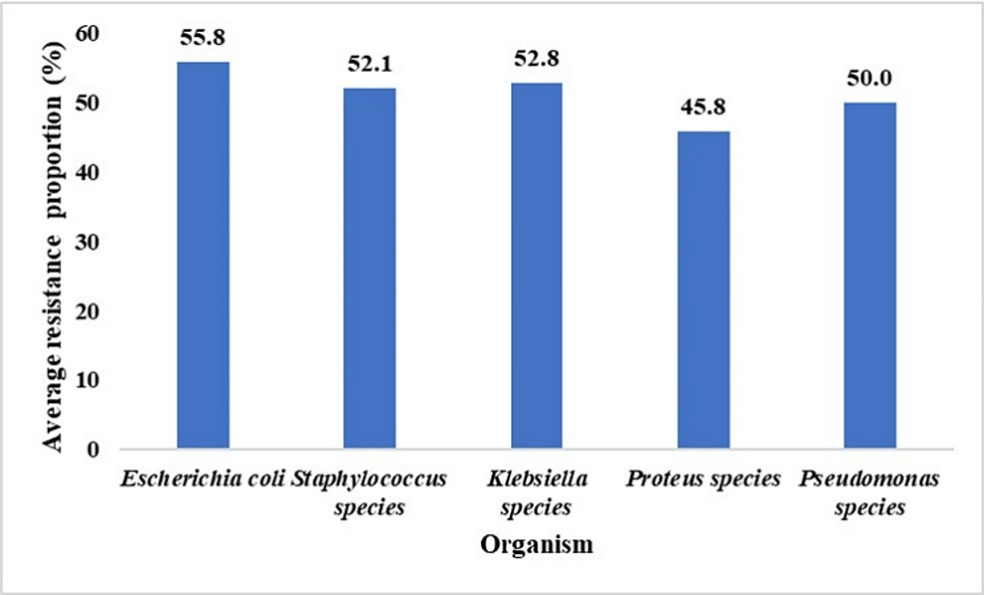
Average proportion of resistance per isolated type of bacteria.

The susceptibility profiles for each antibiotic and bacterium showed variable proportions (Table 4) aggregated per isolate (Figure 3). Regarding the average resistance of each bacterium to all antibiotics, *Escherichia coli* had the highest average (55.8%), and *Proteus* species had the lowest resistance (45.8%) (Figure 3).

**Table 4:** Sensitivity profiles of isolates to different antibacterial agents. *Key*: The proportion of sensitive (S), intermediate (I), and resistant (R) A-dash (-) isolates indicated zero isolates in this category. Of the 52 isolates, 24 were* Escherichia coli*, 11 were *Staphylococcus *species, 14 were *Klebsiella *species = 14, *Klebsiella* species, two were *Proteus *species, and one was *Pseudomonas *species. Ampiclox = ampicillin and cloxacillin; cotrimoxazole = trimethoprim and sulfamethoxazole.

Antibiotic tested	Organism isolated	Sensitivity status N (%)		
		S	I	R
Ampicillin	Escherichia coli	-	-	24 (100.0)
	*Staphylococcus *species	1 (9.1)	2 (18.2)	8 (72.7)
	*Klebsiella *species	-	-	14 (100.0)
	*Proteus *species	-	-	2 (100.0)
	Pseudomonas species	-	-	1 (100.0)
Erythromycin	Escherichia coli	-	5 (20.8)	19 (79.2)
	*Staphylococcus *species	1 (9.1)	4 (36.4)	6 (54.5)
	*Klebsiella *species	-	1 (7.1)	13 (92.9)
	*Proteus *species	1 (50)	-	1 (50.0)
	*Pseudomonas *species	-	-	1 (100.0)
Ampiclox (N=44)	Escherichia coli	-	-	24 (100.1)
	Staphylococcus species	-	-	11 (100.0)
	*Klebsiella *species	-	-	14 (100)
	*Proteus *species	-	-	1 (100.0)
	*Pseudomonas *species	-	-	1 (100.0)
Chloramphenicol	Escherichia coli	12 (50.0)	-	12 (50.0)
	*Staphylococcus *species	6 (54.5)	-	5 (45.5)
	*Klebsiella *species	7 (50.0)	-	7 (50.0)
	*Proteus *species	2 (100.0)	-	-
	*Pseudomonas *species	1 (100.0)	-	-
Ceftriaxone	Escherichia coli	13 (54.2)	3 (12.5)	8 (33.3)
	*Staphylococcus *species	7 (63.6)	2 (18.2)	2 (18.2)
	*Klebsiella *species	12 (85.7)	1 (7.1)	1 (7.1)
	*Proteus *species	1 (50)	1 (50.0)	-
	*Pseudomonas *species	1 (100.0)	-	-
Cotrimoxazole	Escherichia coli	7 (25.5)	-	18 (75.0)
	*Staphylococcus *species	6 (54.5)	-	5 (45.5)
	*Klebsiella *species	7 (50.0)	-	7 (50.0)
	*Proteus *species	-	1 (50.0)	1 (50.0)
	*Pseudomonas *species	-	1 (100.0)	

**Figure 4:**
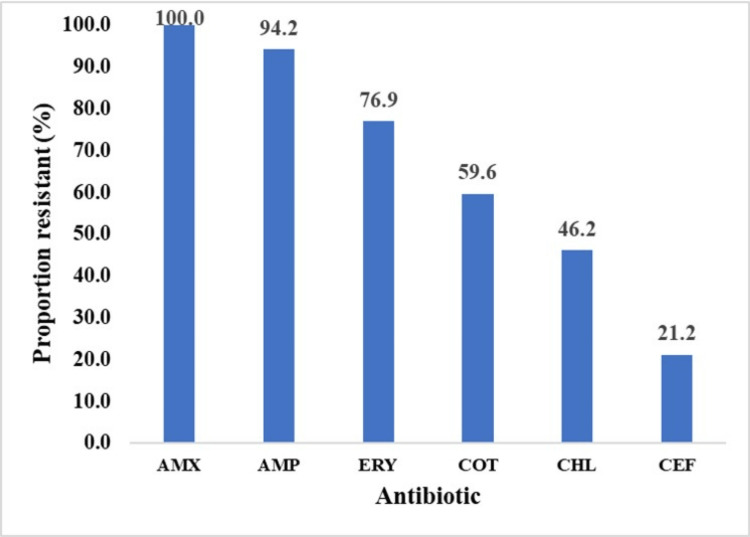
Resistance profiles of the isolated bacteria to the tested antibiotics AMX, ampicillin and cloxacillin; ERY, erythromycin; AMP, ampicillin; CHL, chloramphenicol; CEF, ceftriaxone; COT, trimethoprim and sulfamethoxazole.

Among the tested antibiotics, high resistance to ampicillin (100%) and low resistance to ceftriaxone (21.2%) were observed (Figure 4).

## Discussion

Our study on UTIs in a cohort of children with SCD in Dar es Salaam, Tanzania, revealed that 132 (53%) participants were boys and 117 (47.0%) were between 12 and 15 years of age. The mean age of the participants was 8.79±3.922 years and range 2-15 years. In this study, 24% of children with SCD were prescribed antibiotics, predominantly penicillin V (20.4%), to prevent infections such as pneumococcal sepsis, which are more susceptible to compromised immunity. Prophylactic penicillin V administration from early infancy is advised for those under five years of age to mitigate the risk of invasive pneumococcal disease, with healthcare providers tailoring treatment based on individual needs and monitoring side effects or resistance [20].

We found that the overall prevalence of UTIs was 22.4%. Similar rates of UTI have been observed in other parts of Africa, where the rate of UTI was approximately 26% in children and 20.4 in non-SCD children [7]. The prevalence of UTI in children without SCD attending the clinic at Bagamoyo District Hospital in Tanzania was 16.4% [21], indicating a higher burden of UTI in children with SCD than non-SCD in Tanzania. The prevalence of UTIs caused by dipsticks was 24.8%. However, this difference was not statistically significant (t=-7.41, P=0.459).

UTI occurrence was elevated in the 11-15 age group, suggesting heightened pathogen exposure or hygiene shifts with school attendance. However, UTI risk did not differ significantly across age groups, indicating a uniformly distributed infection risk among children. UTI prevalence was higher in girls than in boys, a phenomenon linked to anatomical differences. Girls have a shorter urethra, making it easier for microorganisms to travel from the gastrointestinal tract to the urinary system, thereby increasing the risk of infection [22]. Although fever is a common symptom associated with UTIs, most study participants did not report fever, suggesting that UTIs in pediatric SCD patients may often present without fever or that fever due to UTI may be underreported in this population [14].

The duration of fever among those who reported symptoms suggests variability in the disease course, which may influence treatment decisions. The fact that most participants did not take any antibacterial medication prior to the study could indicate a low incidence of diagnosed bacterial infections or a possible gap in access to or utilization of healthcare resources.

Leukocyturia was present in one-quarter of cases, suggesting an underlying inflammatory process, although not exclusive to UTIs. Notably, proteinuria was absent in the study group, indicating that renal function in these patients was not significantly impaired by protein leakage, a positive sign of chronic kidney disease risk. For pediatric UTI, the diagnostic performance of dipstick leukocyte esterase and microscopic pyuria varies with urine concentration; therefore, specific gravity should be considered when urinalysis is interpreted [23].

The specific gravity of the urine was 1.030 in 68.7% of the samples, suggesting normal concentration levels in most children. Similarly, urine pH readings fell within the normal range for most participants, providing no immediate cause for alarm but reinforcing the need for regular monitoring, given the known renal complications associated with SCD.

The dipstick test's sensitivity of 17.7% suggests that it identifies only a fraction of actual UTI cases confirmed by culture, raising concerns regarding missed UTIs when used alone. However, its specificity of 84.0% makes it effective in ruling out non-UTI cases. Despite these metrics advocating for their use in preliminary screening, the test's limitations necessitate confirmation by culture for a definitive diagnosis. This study highlights the unreliability of dipsticks for accurate UTI detection in children with SCD, noting discrepancies in prevalence rates compared to culture methods and the risk of false results [24,25]. Consequently, clinicians should not rely solely on the dipstick test for UTI screening, emphasizing the need for corroborative diagnostics.

The most common type of organism isolated was *Escherichia coli *(46.2%), followed by *Klebsiella *species (26.9%), *Staphylococcus *species (21.2%), and Proteus species (3.8%), whereas the least common isolated organism was *Pseudomonas *species (1.8%). Similar isolates have been observed in Nigerian children with SCD [7]. The isolates. *Escherichia coli *is mainly found in the gastrointestinal tract (GI) and UTIs, where the flora is normal; however, UTIs can occur when they enter the urinary system. High rates of Escherichia coli isolation are common in children with SCD [7]. Another study reported higher isolation rates of *Escherichia coli*and *Klebsiella *(64.9% and 18.9%, respectively) [8].

All tested organisms-Escherichia coli, Klebsiella, Proteus, and *Pseudomonas *species-showed 100% resistance to ampicillin, except for *Staphylococcus *(72.7%). Erythromycin resistance was high, especially in Klebsiella (92.9%), *Escherichia coli *(79%), and *Staphylococcus *species (54%). All isolated organisms were resistant to ampiclox (ampicillin and cloxacillin). Resistance to chloramphenicol and cotrimoxazole (trimethoprim and sulfamethoxazole) varied, affecting approximately half of the*Escherichia coli*, *Staphylococcus*, and *Klebsiella *strains. Among the tested antibiotics, ceftriaxone exhibited the lowest resistance. Similar patterns were observed In Maduguri, Nigeria, where uropathogens demonstrated higher sensitivity to third-generation cephalosporins [7,8].

Recommendation

Children with SCD, who are at a higher risk of infections such as UTIs, require vigilant monitoring and possibly a tailored management strategy to ensure prompt diagnosis and treatment, minimizing complications. Routine UTI screening and antibiogram updates are essential to prevent recurrent UTI complications, monitor antibiotic resistance, guide empirical treatment, and provide specific guidelines.

Study limitations

Our study was limited by its single-clinic focus, which potentially affected the generalizability of our findings. The sample size, influenced by multiple regional contexts, may not be accurately reflected in our specific study population. Furthermore, the use of dipstick tests, with their known low sensitivity, underscores the need for more reliable diagnostics, such as culture tests. This cross-sectional design restricts our ability to infer causality and evaluate management strategies over time. We did not fully explore the impact of cultural, behavioral, and socioeconomic factors on UTI risk, nor did we address the monitoring of chronic kidney disease, highlighting the need for an interdisciplinary approach in future studies. Additionally, the restricted range of antibiotics tested may not provide a complete picture of the resistance patterns, necessitating broader antimicrobial testing. Notably, the absence of specific reporting on mixed growths might suggest missed occurrences, emphasizing the need for enhanced scrutiny of sample handling and reporting.

## Conclusions

This study revealed a high prevalence of UTI among children with SCD at the MNH clinic using both dipstick and culture methods. *Escherichia coli *was the most commonly isolated and resistant uropathogen. Compared to ceftriaxone, which was less resistant, high levels of resistance to commonly used antibiotics were noted, with the combination of ampicillin and cloxacillin showing the highest resistance. Girls were more likely to be UTI-positive than boys.
